# Sleep Difficulties, Sleep Duration, and Sleeping Place in Early Childhood: A Longitudinal Study on Stability and Inter-Relations from 1 to 5 Years

**DOI:** 10.3390/pediatric18030068

**Published:** 2026-05-14

**Authors:** Tanja Poulain, Juliane Ludwig, Nico Grafe, Andreas Merkenschlager, Wieland Kiess

**Affiliations:** 1 LIFE Child, Leipzig Research Center for Civilization Diseases, Leipzig University, 04103 Leipzig, Germany; juliane.ludwig@medizin.uni-leipzig.de (J.L.); nico.grafe@medizin.uni-leipzig.de (N.G.); wieland.kiess@medizin.uni-leipzig.de (W.K.); 2 Department of Women and Child Health, Hospital for Children and Adolescents and Center for Paediatric Research (CPL), Leipzig University, 04103 Leipzig, Germany; andreas.merkenschlager@medizin.uni-leipzig.de; 3 German Center for Child and Adolescent Health (DZKJ), Partner Site Leipzig/Dresden, 04103 Leipzig, Germany

**Keywords:** children, stability, longitudinal, sleep difficulties, sleep duration, sleeping place

## Abstract

Background/Objectives: This longitudinal study examined the association between sleep duration, sleep difficulties, and sleeping place at one year (t1) and corresponding characteristics at 4/5 years of age (t2). Methods: Data were collected from 2018 to 2021 (t1) and from 2021 to 2024 (t2) in the LIFE Child cohort study conducted in Leipzig, Germany. Parents completed the Brief Infant Sleep Questionnaire at t1 and the Child Sleep Habits Questionnaire at t2. Associations between sleep characteristics at t1 and t2 were estimated using linear and logistic regression models. All associations were adjusted for child sex, age at t2, and maternal education. Results: The analyses showed significant associations between shorter sleep durations, later sleep onset times, more frequent nightly awakenings and bed sharing and room sharing at t1 and more sleep difficulties at t2. A shorter sleep duration at t2 was predicted by shorter sleep and more parent-perceived sleep difficulties at t1. Bed sharing and room sharing at t1 were significantly associated with a lower probability of sleeping alone at t2. Conclusions: These results indicate that sleep duration, sleep difficulties, and sleeping places are already stable in early childhood.

## 1. Introduction

Sufficient sleep and good sleep quality are basic requirements for healthy child development. Poor sleep, in contrast, is associated with reduced health and performance [1].

In Germany, children aged 1 year slept an average of 13 h per day in 2003–2006, while 4-year-olds slept around 11 h [2]. Furthermore, around 23% of parents reported sleep difficulties in 0- to 2-year-olds. For 3- to 10-year-olds, it was 16% [2]. In our previous German study, for which data were collected between 2011 and 2015, children aged 4 to 9 years slept an average of 10 h per night, and the prevalence of sleep difficulties was estimated at 20% [3]. Some studies suggest that early sleep difficulties persist until childhood [4,5] and adolescence [6]. Similar trends were observed for sleep duration [7].

From a transactional perspective [8], child development emerges from dynamic, bidirectional interactions between the child and the caregiving environment over time. Applied to sleep, this implies that children’s sleep patterns are shaped by ongoing exchanges between individual characteristics and environmental factors such as caregiving practices and sleep arrangements. Several previous studies suggest that the sleep quality of children is influenced by sleep habits, e.g., bedtimes and bedtime routines [9], and that these habits are linked to family settings and parental attitudes [10]. Parental attitudes, early sleep habits, and sleep–wake rhythmicity show a degree of stability across early childhood and may therefore contribute to the persistence of sleep difficulties over time [7].

The sleeping place represents an especially important and proximal environmental factor as it directly structures sleep-related interactions. At the same time, it may constitute an integral component of the sleep process itself, as it shapes how children initiate and maintain sleep and how caregivers respond to nocturnal awakenings. A distinction is often made between sleeping in one’s own bed and room, sleeping in one’s own bed in the parents’ room (room sharing), and sleeping in the parents’ bed (bed sharing) [11]. While bed sharing is viewed positively in some countries, it is considered problematic in Europe, North Asia and North America [12,13]. A main reason for this is that bed sharing is considered a risk factor for sudden infant death syndrome in the first year of life [11]. Room sharing, on the other hand, is recommended in the first 6 to 12 months [11,14]. From then on, sleeping in one’s own bed and room is considered the best in Western industrialized societies and in some sleep instruments developed there (e.g., [15,16]). However, whether the sleeping place is associated with children’s sleep is controversial [12,17].

The primary goal of this study was to investigate whether sleep duration and sleep difficulties at 4/5 years of age were predicted by sleep characteristics at 1 year of age, including sleep duration, difficulties and children’s sleeping place. The secondary goal was to assess the stability of sleeping places from age 1 to 4/5 years. Unlike previous longitudinal studies conducted in North America [4], Asia [5,7], or other European countries [6], which primarily focused on young infants and children [4,5,7], or adolescents [6], the sample in this study included children from Germany spanning a wide age range between the survey time points. We hypothesized significant associations between shorter sleep durations, later sleep onset times, more night awakenings, and more sleep difficulties at 1 year and more sleep difficulties as well as shorter sleep durations at age 4/5 years. Additionally, we expected a persistence of sleeping places from 1 to 4/5 years of age.

## 2. Materials and Methods

### 2.1. Participants

Data were collected between 2018 and 2024 within the LIFE Child study, a cohort study conducted at Leipzig University [18]. LIFE Child investigates the development of healthy children and adolescents. All children not suffering from any chronic, chromosomal, or syndromic disease are eligible for participation. They are recruited until the age of 16 years and mainly come from the city of Leipzig. All children and their parents are invited to attend annual follow-up visits. The study program was designed in accordance with the Declaration of Helsinki and was approved by the Ethics Committee of the Medical Faculty of Leipzig University (477/19-ek, 3 December 2020).

For the present analyses, all children for whom information on sleep duration, sleep difficulties, and sleeping place was available at 1 year (t1, data collected between 2018 and 2021) and at 4/5 years (t2, data collected between 2021 and 2024) were considered. Of 563 children whose parents had provided information on their children’s sleep at t1, 101 (18%) also participated at t2 (56 boys, 45 girls). Mean age at t1 was 1.0 years (sd = 0.06, range 0.9–1.2). At t2, mean age was 4.6 (sd = 0.44, range 4.0–5.2).

Some of the data was collected during the COVID-19 pandemic. However, measurements were only taken when general restrictions were low, parents were able to go to work, and children could be cared for in daycare centers. There were no significant differences in the variables depending on whether data were collected during the pandemic period (defined as April 2020 to the end of 2022) or outside of it (all *p* > 0.273). Therefore, the timing of data collection (during the COVID-19 pandemic or not) was not considered as a covariate.

### 2.2. Instruments

#### 2.2.1. Sleep Characteristics

Sleep characteristics at t1 were assessed using the Brief Infant Sleep Questionnaire BISQ [19]. The questionnaire comprises 10 questions on children’s sleep duration, sleep difficulties, and sleeping place. We included the questions on nocturnal sleep duration, daytime sleep duration, number of nightly awakenings, nocturnal sleep-onset time, sleeping place, and parent-perceived sleep difficulties. Other questions were not included in the analyses either because they were not central to the research question (infant-specific sleep positions and bedtime routines) or due to substantial (>25%) missing data (duration of nightly awakenings and sleep onset latency). Regarding sleeping place, the response options room sharing, bed sharing, and sleeping alone in one’s own room were included. Regarding parent-perceived sleep difficulties, we distinguished no difficulties and mild/strong difficulties. For all other questions, we analyzed the times/durations/numbers as indicated by parents.

At t2, sleep duration, sleep difficulties, and sleeping place were measured using the Children’s Sleep Health Questionnaire CSHQ [15,16], completed by a parent. This questionnaire consists of 33 questions on several sleep difficulties (e.g., problems falling asleep, anxiety when sleeping alone). Each question is answered on a 3-point Likert scale, and item responses are summed to a CSHQ total score, with higher values indicating more sleep difficulties. Furthermore, parents indicated children’s sleep duration (night and day combined). For a more detailed analysis of sleeping place at t2, two items of the CSHQ (sleeping alone in one’s own room and bed sharing with parents or siblings) were investigated separately. For each of these variables, the response option “usually” was considered to indicate the corresponding sleeping place. Room sharing was not assessed in the CSHQ.

In sum, sleep duration was assessed based on nocturnal and daytime sleep duration at t1 and total sleep duration at t2. Sleep difficulties were indexed by the number of nightly awakenings, nocturnal sleep onset, and parent-perceived sleep difficulties at t1, and by the CSHQ total score at t2. Sleeping place was captured via room sharing, bed sharing, and sleeping alone in one’s own bed at t1, and via bed sharing and sleeping alone in one’s own room at t2.

#### 2.2.2. Covariates

Child age (at t2), sex, and maternal education (at t2) were included as covariates. Maternal education was assessed based on their highest school degree and their highest professional education. Information on both was combined to a score ranging from 1 (no school degree) to 7 (university degree), where higher values indicate higher education [20].

### 2.3. Statistical Analysis

Statistical analysis was performed in R version 4.5 [21]. Variables were described in terms of means and standard deviations (for continuous variables) or absolute and relative frequencies (for categorical variables). To compare the final sample (n = 101) with the sample of children for whom information on sleep was available at t1 but not at t2 (n = 462), we performed analyses of variance (for continuous sociodemographic and sleep-related variables) or chi squared tests (for categoriacal variables), with “t2 available” (yes versus no) as the independent variable. Associations between sleep variables at t1 (independent variables) and sleep duration, CSHQ total score (indicating sleep difficulties), and sleeping place at t2 (dependent variables) were assessed using linear or logistic regression analyses. All associations were adjusted for the mentioned covariates. We report both raw and adjusted *p*-values. Adjustment for multiple comparisons was performed using the Benjamini–Hochberg procedure to control the false discovery rate (FDR). To assess multicollinearity, we examined variance inflation factors (VIFs). All VIF values were below 5, indicating no evidence of problematic multicollinearity.

## 3. Results

### 3.1. Characteristics of the Study Population

Characteristics of the study population at t1 and t2 are summarized in Table 1. The average CSHQ score at t2 was 47.8. The average total sleep durations were 13 and 11 h at t1 and t2, respectively. Regarding the sleeping place at t1, room sharing was reported most frequently (42%), while bed sharing and sleeping alone were each reported by 24% of parents. At t2, 48% of children usually slept in their own bed in their own room and 33% usually slept in their parents’ or siblings’ bed.

Children of this sample did not differ significantly from children of the LIFE Child cohort whose parents provided information on their children’s sleep at t1 but not t2 (n = 462), neither in terms of maternal education nor in terms of sleep (sleep duration night, sleep duration day, sleeping place, sleep onset time, number of night awakenings, parent-perceived sleep difficulties), all *p* > 0.05.

### 3.2. Associations Between Sleep Variables at Age 1 and 4/5 Years

**Figure 1:**
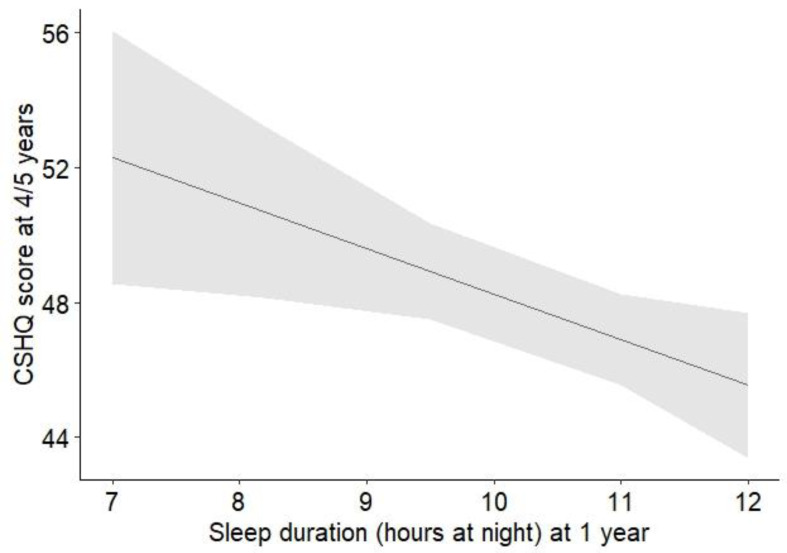
Effect plot illustrating the association (+95% CI) between sleep duration at t1 (1 year of age) and CSHQ scores at t2 (age 4/5 years). Higher CSHQ scores indicate more sleep difficulties.

Longer nocturnal sleep duration at the age of 1 year was significantly associated with a lower CSHQ score at age 4/5 years. With each additional hour of nocturnal sleep at t1, the CSHQ score at t2 decreased by 1.35 points (b = −1.35, *p* = 0.015, see Figure 1). Furthermore, the analyses revealed significant associations between room sharing and bed sharing at t1 and higher CSHQ scores at t2. In both cases (room sharing and bed sharing), children had CSHQ scores that were more than half a standard deviation higher than those of children who slept in their own bed and room (b = 3.20 and 4.19, *p* = 0.033 and 0.013, respectively). Later sleep onset times at t1 (b = 1.50, *p* = 0.049) and a higher number of nightly awakenings at t1 (b = 0.88, *p* = 0.030) were also significantly associated with higher CSHQ scores at t2. Parent-perceived sleep difficulties at t1, in contrast, were not significantly associated with CSHQ scores at t2 (see Table 2). After adjustment for multiple testing, none of the mentioned associations remained statistically significant (see Table 2).

**Figure 2:**
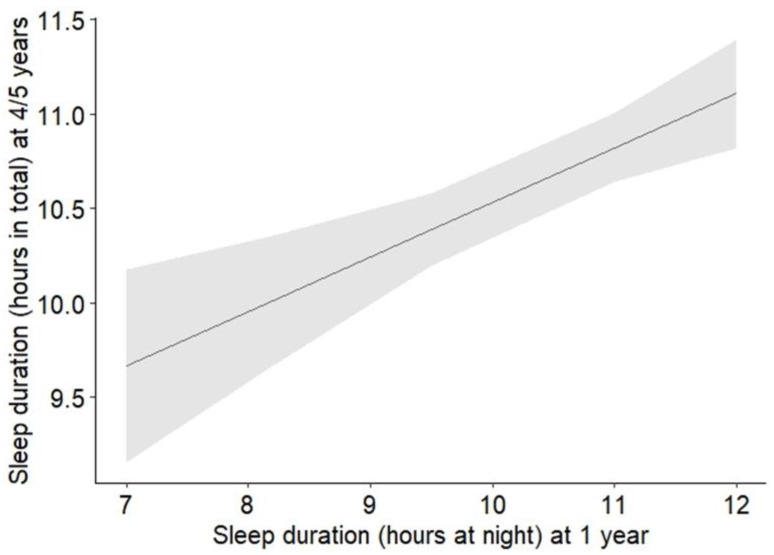
Effect plot illustrating the association (+95% CI) between sleep duration at t1 (1 year of age) and sleep duration at t2 (age 4/5 years).

Regarding sleep duration, longer nocturnal sleep durations at t1 were significantly associated with longer sleep durations at t2. With each additional hour of nocturnal sleep at t1, the sleep duration at t1 increased by 0.29 h, i.e., 17 min (b = 0.29, *p* < 0.001; see Figure 2). This association remained statistically significant after adjusting for multiple testing (*p* = 0.005). The analyses also revealed a significant association between parent-reported sleep difficulties at t1 and shorter sleep durations at t2 (b = −0.43, *p* = 0.012). In more detail, children whose parents reported sleep difficulties in their children at t1 slept on overage 10 h and 20 min at t2, compared to 10 h and 45 min in children whose parents did not report sleep difficulties at t1. However, this association did not remain statistically significant after adjusting for multiple testing (*p* = 0.063). The other sleep difficulties at t1, as well as sleeping place, were not significantly associated with sleep duration at t2 (see Table 2).

Overall, the confidence intervals were rather large (see Table 2), indicating limited precision of the estimated effects.

Compared with children sleeping in their own bed and room, those sleeping in their parents’ room or bed at t1 slept significantly less frequently in their own room and bed at t2 (OR = 0.14 (95% CI 0.04–0.50) and 0.03 (95% CI 0.007–0.17), *p* = 0.003 and <0.001, respectively). For example, of those children sleeping in their parents’ bed at 1 year, only 17% slept in their own room and bed at 4/5 years, compared to 83% of those sleeping in their own bed and room already at 1 year. These associations remained statistically significant after adjusting for multiple testing (*p* = 0.039 for room sharing and 0.002 for bed sharing at t1).

Similarly, in children sleeping in their parents’ bed at t1, bed sharing was significantly more frequent at t2 (58%, OR = 7.30 (95% CI 1.86–28.70), *p* = 0.004) than in children sleeping in their own bed and room at 1 year (17%). This association lost statistical significance after adjusting for multiple testing (*p* = 0.053). The association between room sharing at t1 and bed sharing at t2 was not significant (OR = 2.9 (95% CI 0.83–10.44), *p* = 0.095).

## 4. Discussion

The primary goal of this study was to investigate associations between sleep difficulties, sleep durations, and sleeping place at 1 year of age and sleep duration and difficulties at age 4 to 5 years. Our analyses revealed significant associations between shorter nocturnal sleep durations, room sharing, bed sharing, later sleep onset times, and higher number of nightly awakenings at 1 year and more sleep difficulties at age 4/5 years. Although these associations did not remain statistically significant after adjusting for multiple testing, the findings suggest continuity in sleep-related behaviors and difficulties across early childhood. However, given that sleep constructs were assessed using different instruments at the two time points (BISQ at 1 year and CSHQ at 4–5 years), these results should be interpreted as reflecting related but not identical aspects of sleep rather than strict longitudinal stability of the same construct. Accordingly, differences in measurement approaches may also account for the lack of a significant association between parental perceptions of sleep difficulties at 1 year and CSHQ scores at 4–5 years, as reduced comparability across instruments may have attenuated observed associations. In addition, as both assessments were based on parent-reported measures, shared method variance may have contributed to an overestimation of longitudinal associations due to consistent reporting tendencies across time points. Nevertheless, the overall pattern is broadly consistent with previous evidence indicating relative stability of early sleep patterns [4,5].

Regarding sleep duration, the averages reported at both time points were comparable with the sleep durations reported in previous German samples [2,3]. Furthermore, our results confirm previous reports on associations between sleep duration at 1 and 4 years [7]. They, therefore, suggest that it could already become apparent in early childhood whether someone is more of a short or long sleeper. A significant association between parents’ perception of child sleep difficulties at 1 year and shorter sleep durations 3 to 4 years later supports the assumption that sleep difficulties are stable in early childhood (even if the association lost statistical significance after adjusting for multiple testing).

The secondary goal of this study was to assess stability in sleeping places from 1 to 4/5 years of age. At the age of 1 year, nearly 3 quarters of children slept either in their own bed and room or in their parents’ room and, therefore, followed the current recommendations regarding sleeping place [11,14]. The finding that children who already sleep alone at the age of one year also do so at the age of 4/5 years is not surprising. More interestingly, the analyses revealed that bed sharing is not a phenomenon of the first year of life, but is still observed at the age of 4/5 years, especially in children in whom this was already the case at the age of 1 year. Unfortunately, we did not assess whether bed sharing represented a problem for parents (or children). If yes, the finding suggests stability in problematic sleep habits and/or the difficulty of changing sleeping places throughout childhood. If not, however, persistence of sleeping places might simply reflect the strength of parental attitudes. While bed sharing is considered problematic by some parents, other parents do not see it as problematic, but voluntarily choose to share their bed with their child [13]. In this context, it should generally be questioned whether room or bed sharing should be considered problematic in sleep instruments or rather be surveyed and reported more neutrally.

Following the transactional perspective on child development [8], the observed associations between early sleep characteristics and later sleep outcomes may reflect the stability of child–environment transactions, in which early-established sleep-related interactions gradually consolidate over time. In particular, practices such as room sharing or bed sharing may become embedded within family routines and expectations, potentially contributing to the persistence of sleep-related behaviors across early childhood.

Our findings highlight the importance of early identification of sleep difficulties in pediatric care and the potential value of early guidance for caregivers regarding sleep hygiene and sleeping places.

### Strengths and Limitations

This study used a longitudinal design to assess associations between early sleep difficulties and routines and sleep difficulties in later childhood. Furthermore, sleep was described using several behavioral measures, allowing for a thorough and detailed analysis. However, some limitations have to be acknowledged. The sample size was small, minimizing the statistical power and the model stability. In addition, all participants came from one city in Germany, and children from families with a lower socio-economic position were underrepresented. Therefore, the results may only be applicable to the general population to a limited extent. Instead of using objective sleep measures, e.g., actigraphy, we relied on parental reports, which are subjective and might be biased (e.g., social desirability). Moreover, shared reporting bias across time points could artificially inflate observed associations. Another limitation is that sleep characteristics were assessed using different instruments at t1 and t2. Although both measures capture related aspects of sleep difficulties, differences in operationalization (e.g., regarding sleeping place) may limit the direct comparability of the constructs across time and could have introduced measurement-related variance into the observed associations. Furthermore, potentially relevant confounders, e.g., parenting practices or child temperament, were not assessed and, therefore, could not be included as covariates. Finally, the absence of longitudinal modeling techniques limited the depth of analysis.

## 5. Conclusions

Overall, the results of this study indicate that sleep duration, sleep difficulties and sleeping places at 1 year of age are associated with sleep difficulties and sleeping places at later childhood. Future research would benefit from larger samples of children, the use of more objective measures, and more advanced modeling techniques.

## Figures and Tables

**Table 1:** Sociodemographic and sleep characteristics of the study population at t1 and t2.

		t1 (1 Year)	t2 (4 to 5 Years)
Years of assessment	Range	2018–2021	2021–2024
Years between t1/t2	M (sd)	3.7 (0.46)	
Age	M (sd)	1.0 (0.06)	4.6 (0.44)
Sex	N (%) male	56 (55%)	
	N (%) female	45 (45%)	
Maternal education	M (sd)	5.9 (1.27)	6.1 (1.29)
	N (%) highest ^a^	37 (36%)	63 (62%)
CSHQ score	M (sd)	-	47.8 (5.81)
Sleep duration	M (sd)	-	10.6 (0.86)
Sleep duration night	M (sd)	10.3 (1.08)	-
Sleep duration day	M (sd)	2.3 (0.68)	-
Sleeping place	N (%) own room/bed	24 (24%)	48 (48%)
	N (%) room sharing	42 (42%)	
	N (%) bed sharing	24 (24%)	33 (33%)
Sleep onset time (h:min)	M (sd)	19:40 (0:46)	-
N night awakenings	M (sd)	2.7 (1.5)	-
Parent-perceived sleep difficulties	N (%) yes	35 (35%)	-

^a^ highest score (7) indicates university degree.

**Table 2:** Associations between sleep characteristics at t1 and sleep difficulties and sleep duration at t2.

		Sleep Characteristics at t2				
	CSHQ total score			Total sleep duration		
Sleep characteristics at t1	b (95%CI)	*p*	*p* adj.	b (95%CI)	*p*	*p* adj.
Sleep duration night	−1.35 (−2.44, −0.27)	0.015	0.070	0.29 (0.14, 0.43)	<0.001	0.005
Sleep duration day	0.67 (−1.07, 2.41)	0.445	0.703	−0.01 (−0.26, 0.24)	0.934	0.971
Room sharing ^a^	3.20 (0.27, 6.13)	0.033	0.103	0.25 (−0.17, 0.66)	0.245	0.459
Bed sharing ^a^	4.19 (0.88, 7.49)	0.013	0.067	0.37 (−0.11, 0.84)	0.127	0.305
Sleep onset time	1.50 (0.001, 2.99)	0.049	0.142	−0.04 (−0.26, 0.17)	0.658	0.858
Number night awakenings	0.88 (0.09, 1.67)	0.030	0.103	−0.02 (−0.14, 0.09)	0.707	0.902
Parent-perceived sleep difficulties (mild/strong) ^b^	1.82 (−0.64, 4.27)	0.145	0.322	−0.43 (−0.78, −0.10)	0.012	0.063

All associations are adjusted for sex, age at t2, and maternal education at t2. ^a^ Reference = sleeping in one’s own room and bed; ^b^ Reference = no sleep difficulties.

## Data Availability

Data cannot be shared publicly because there exist ethical restrictions. The LIFE Child study is a study collecting potentially sensitive information. Publishing data sets is not covered by the informed consent provided by the study participants. Furthermore, the data protection concept of LIFE requests that all (external as well as internal) researchers interested in accessing data sign a project agreement. Researchers that are interested in accessing and analyzing data collected in the LIFE Child study may contact the data use and access committee (forschungsdaten@medizin.uni-leipzig.de).
